# Impact of a Novel Electronic Medical Record–Integrated Electronic Form (Provider Asthma Assessment Form) and Severe Asthma Algorithm in Primary Care: Single-Center, Pre- and Postobservational Study

**DOI:** 10.2196/74043

**Published:** 2025-06-25

**Authors:** Matheson L McFarlane, Alison Morra, Delanya Podgers, David Barber, M Diane Lougheed

**Affiliations:** 1 Asthma Research Unit, Kingston Health Sciences Centre, 76 Stuart Street, Kingston, ON, K7L 2V7, Canada, 1 6474608012; 2 Department of Medicine, Division of Respirology, Queen's University, Kingston, ON, Canada; 3 Kingston General Hospital, Kingston Health Sciences Centre, Kingston, ON, Canada; 4 Canadian Primary Care Sentinel Surveillance Network, Kingston, ON, Canada; 5 Department of Family Medicine, Queen's University, Kingston, ON, Canada

**Keywords:** asthma, severe asthma, diagnosis, pulmonary function tests, guideline implementation, digital tools, electronic forms, best practice, knowledge translation

## Abstract

**Background:**

Despite national asthma care guidelines, care gaps persist between best-practice and clinical practice, contributing to poor health outcomes. The Provider Asthma Assessment Form (PAAF) is an electronic asthma management and Knowledge Translation tool with an embedded decision support algorithm for severe and/or uncontrolled asthma, designed to support evidence-based asthma management.

**Objective:**

In this study, we aimed to document baseline asthma practice patterns and determine whether the broader intervention of PAAF integration into a primary care electronic medical record (EMR) improves evidence-based asthma diagnosis and management.

**Methods:**

We performed a single-center pre- and postobservational study at an academic Family Health Team in Kingston, Ontario, Canada. Retrospective baseline data were collected for 2 years prior to PAAF implementation from January 2018 to December 2019. Prospective postintervention data were collected from October 2022 to July 2024. A validated adult asthma EMR case definition was applied to EMR data to identify suspected or objectively confirmed asthma cases for both datasets, on which detailed manual chart abstractions were performed. A data extraction was performed for completed PAAFs.

**Results:**

There were 230 patients in the retrospective baseline and 143 patients in the postimplementation cohort. Overall, 31.3% (n=72) of patients at baseline versus 23.8% (n=34) at postimplementation had confirmed asthma. There were significantly more pulmonary function tests requested after the implementation of the PAAF (postimplementation: n=70, 49%; baseline: n=71, 30.9%; *P*<.001). A significantly higher percent of postimplementation patients were on single inhaler controller and reliever therapy (postimplementation: n=31, 21.7%; baseline: n=2, 0.9%; *P*<.001), inhaled corticosteroid/long-acting β-2 agonist therapy (postimplementation: n=36, 25.2%; baseline: n=34, 14.8%; *P*=.01), and inhaled corticosteroid if their asthma was uncontrolled (postimplementation: n=69, 62.2%; baseline: n=100, 43.5%; *P*=.002). Barriers were significantly more commonly addressed after implementation (postimplementation: n=24, 16.8%; baseline: n=11, 4.8%; *P*<.001). A significantly higher average number of asthma control parameters was documented when the PAAF was used (PAAF: mean 5.4, SD 1.9; manual chart abstraction: mean 2.3, SD 1.2; *P*<.001). Care as assessed by key Primary Care—Asthma Performance Indicators showed improvement in the postimplementation cohort, which did not reach statistical significance.

**Conclusions:**

The multifaceted intervention of implementing the PAAF in this primary care practice was associated with improved documentation of diagnosis status and asthma control parameters and improved adherence with evidence-based recommendations for care, such as the use of pulmonary function tests and addressing barriers to effective asthma management. However, uptake was low, and key asthma care gaps were still common. Future directions should involve evaluating the impact of the PAAF on care and outcomes after widespread implementation in primary care settings and investigating methods to increase user uptake of the PAAF.

## Introduction

### Background

Asthma is a common chronic respiratory disease contributing to a significant health care burden on both the individual and society [1,2]. It is defined as an inflammatory condition of the airways, characterized by episodic or persistent symptoms, such as dyspnea, wheezing, and cough, and is associated with variable airflow limitation and airway hyperresponsiveness [3]. Recommendations for best-practice asthma diagnosis and management are disseminated to primary care providers through the publication of clinical guidelines. In Canada, the Canadian Thoracic Society (CTS) evidence-based guidelines state that best-practice diagnosis is based on a compatible clinical history and objective evidence of asthma or a specialist diagnosis [3]. Effective management includes assessment of asthma control, patient self-management education, identifying triggers and environmental controls, and appropriate pharmacological therapy [3].

However, difficulties integrating clinical guidelines into the process of care have contributed to a number of key asthma care gaps in the primary care setting, causing a separation between best-practice and current clinical practice [3-7]. Several key asthma care gaps have been identified, primarily concerning objective diagnosis, continued monitoring with pulmonary function tests (PFTs), addressing asthma control, and delivering patient self-management education [5,8-11]. Furthermore, individuals with severe asthma (SA; 5%‐10% of patients with asthma) account for a disproportionate 50% of asthma-related health care costs [6]. Hence, in a recent position statement, the CTS highlighted a greater need for adequate recognition and management of individuals with SA and/or uncontrolled asthma, including specialist referral and consideration of biologic therapies when appropriate [6].

Consequently, the introduction of Knowledge Translation (KT) initiatives and tools to aid in adopting clinical guidelines in primary care practice is warranted [4]. Electronic medical records (EMRs) provide a unique opportunity at the point-of-care to integrate novel electronic tools (e-Tools), as they facilitate sentinel surveillance, performance benchmarking, and quality improvement [12]. Several validated and beneficial e-Tools have been researched. However, limitations exist with their use, including limited user uptake beyond the scope of research, the absence of standardized data elements, and limited involvement of end users throughout the design process [12-16].

An e-Tool designed for primary care EMRs that addresses the limitations of current e-Tools may have a significant impact on primary care asthma diagnosis and management and prompt adherence to best-practice guidelines. The Provider Asthma Assessment Form (PAAF) is a novel, point-of-care asthma management tool with an embedded SA algorithm and clinical decision support [17]. It was adapted from the Lung Health Foundation’s Asthma Care Map for primary care (paper tool), is congruent with current CTS guidelines, and incorporates Primary Care—Asthma Performance Indicators (PC-APIs) and Pan-Canadian Respiratory Standards Initiative for Electronic Health Records (PRESTINE) standardized asthma data elements [3,12,17,18]. It was developed by the Asthma Research Unit at Queen’s University in collaboration with primary care providers and has been integrated into the OSCAR primary care EMR at an academic Family Health Team (FHT) in Kingston, Ontario. The ultimate aim of this initiative is to address the major key asthma care gaps in primary care and promote adherence to best-practice guidelines through the introduction of a novel e-Tool to aid primary care providers in their practice.

### Objectives

We conducted a comprehensive evaluation of the integration of the PAAF into a primary care EMR to justify the widespread implementation of this form in primary care EMRs and other practice settings. Principally, we aimed to assess the ability of the PAAF’s implementation to address several key asthma care gaps in asthma diagnosis, management, and surveillance: underuse of objective measures of lung function to diagnose asthma [19-21], inconsistent documentation of asthma diagnosis in primary care EMRs [22], and underrecognition and suboptimal management of SA and/or uncontrolled asthma [6]. The primary objective was to evaluate the impact of the PAAF’s implementation on the use of objective lung function measures to confirm asthma diagnoses, documentation in the EMR of asthma diagnosis status (suspected, confirmed, or excluded), and referral of individuals with suspected or confirmed SA to a specialist.

## Methods

### Ethical Considerations

The study received ethics approval from the Queen’s University Health Sciences & Affiliated Teaching Hospitals’ Research Ethics Board (HSREB #6036789). This study involves using existing data from the Canadian Primary Care Sentinel Surveillance Network (CPCSSN) project (HSREB# 19809). CPCSSN has a data-sharing agreement with the center where this study was conducted. Passive consent was used, with all patients having an opportunity to explicitly opt out of the study. All data were deidentified.

### Study Design

A pre- and postobservational study was conducted at an FHT in Kingston, Ontario. The study design consisted of 2 cohorts of patients: a retrospective baseline cohort, consisting of patients who had an asthma visit before implementation of the PAAF, and a postimplementation cohort, consisting of patients with a visit for asthma after integration of the PAAF into the OSCAR EMR at an FHT. An asthma visit was defined as an FHT encounter where asthma was suspected, diagnosed, managed, or treated either at an in-person or telephone appointment. Case identification was accomplished by applying the newly validated adult asthma case definition for primary care EMRs to CPCSSN-deidentified EMR data for the FHT [22]. This case definition identified adult patients (≥18 years of age) with either suspected or confirmed asthma and a documented encounter during the defined study period. This validated case definition has a sensitivity of 81% and specificity of 96% and is based on *International Classification of Diseases, Ninth Revision* (*ICD-9*) codes for asthma billing [22]. A single abstractor completed detailed manual chart abstraction on identified cases. A programmed data extraction from the FHT EMR was subsequently performed for all PAAFs that were used within the study period.

**Figure 1.**
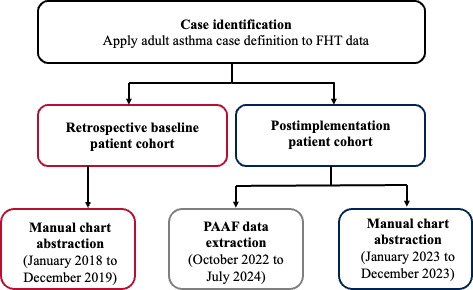
Patient cohort identification methodology. FHT: Family Health Team; PAAF: Provider Asthma Assessment Form.

The retrospective baseline cohort included all asthma visits in a 2-year period between January 2018 and December 2019 prior to the COVID-19 pandemic to ensure that baseline data were representative of usual care. The postimplementation period was set as January 2023 to December 2023 (1 calendar year) for manual chart abstractions. Due to barriers faced with implementation (technological difficulties with the form’s functionality in the EMR from May to July 2023) and challenges with user uptake, the period for completed PAAF data extraction was delayed until October 2022 to July 2024. The sample derivation methodology is summarized in Figure 1.

### Intervention and Implementation

The PAAF is a web-based electronic form with direct access from the patient dashboard in the OSCAR EMR at the FHT. Patient demographics such as date of birth and chart number are automatically populated when the electronic form is opened in a patient chart. It contains 12 sections pertaining to the diagnosis, family history, smoking history, asthma severity, occupational history, respiratory medications, asthma control, care, management and referrals, asthma action plan, asthma control zone, pulmonary function tests, and assessment tools. Select sections include interactive features, such as a calculation of smoking pack-years and a determination of “yes” or “no” if the patient’s asthma is controlled, based on the CTS control criteria. The SA algorithm feature consists of a decision-support prompt appearing at the top of the form once relevant sections have been completed. Decision support prompts include suggesting confirmation of an asthma diagnosis through objective measures, tapering inhaled corticosteroid (ICS) medication if the patient’s asthma is in control, addressing reasons for poorly controlled asthma, and considering a referral to a specialist for SA and/or uncontrolled asthma.

Various methods of implementation were used throughout the PAAF study period from October 2022 to July 2024. Initial implementation was conducted by a site champion, a first-year family medicine resident at the FHT. The PAAF was initially introduced by the site champion to attending and resident physicians through a presentation and via email. Subsequent implementation efforts throughout the course of the study period included a reminder poster in the FHT clinic team rooms about the PAAF and how to access it in OSCAR.

A summary email and an attached 1-page handout detailing the purpose of the PAAF and how to use it were also sent to all FHT providers via email twice throughout the study period. Additionally, the study team collaborated with a nursing student community-based project group. The nursing students were given a summary presentation about asthma, the key asthma care gaps, and how the PAAF can be used in clinical practice. They participated in a tutorial and live demonstration of the PAAF within the OSCAR EMR. The students then used the form during telephone appointments of patients with asthma with supervision from an instructor.

Finally, implementation strategies for the PAAF included educational presentations at Family Medicine Grand Rounds. In both July 2023 and July 2024, the study team gave presentations to incoming first-year family medicine residents during their orientation period. Additional presentations were given throughout the year to upper-year residents on rotation at the FHT.

### Manual Chart Abstraction and Data Extraction

#### Overview

Detailed manual chart abstractions were completed by a single abstractor for adult asthma cases identified in both study cohorts. Chart abstractions were completed using a PAAF manual chart abstraction form consisting of all data elements within the PAAF to ensure consistency and accuracy of data entry. In total, 140 data elements were collected from patient charts. Chart abstractions were internally validated by a registered nurse knowledgeable in asthma management, with guidance from a respirologist. All data collected were deidentified. Data elements included patient demographics, comorbidities, classification of diagnosis status, asthma control, health services use, respiratory medications, smoking status, and care, management and education. Medication classification and ICS dosage rating (low, medium, or high dose) were guided by the Lung Health Foundation’s respiratory medication reference document and CTS dosing categories [3]. Additionally, a programed data extraction was performed for all PAAFs partially or fully completed throughout the study duration.

#### Classification of Confirmed Versus Suspected Asthma

Each patient’s asthma diagnosis was classified as confirmed, suspected, or excluded based on whether or not PFTs were ever present in the patient EMR chart, which met the criteria for asthma based on CTS guidelines [3]. Suspected asthma was defined as a compatible clinical history without objective evidence of asthma. Confirmed asthma included a compatible clinical history and objective evidence of asthma on PFTs or a specialist diagnosis (ie, respirologist and allergist). A specialist diagnosis was accepted based on the CTS Severe Asthma Position Statement recommending referral to a specialist when asthma is difficult to confirm based on objective measures (eg, due to the presence of fixed obstruction) [6] and the CTS’ PRESTINE expert Delphi panel [17].

Although an empiric trial of controller therapy can be used in the diagnosis of asthma, particularly when access to objective testing is limited or unavailable (eg, during the COVID-19 pandemic), the Choosing Wisely Canada recommendations (consolidated and written by the CTS) emphasize to not continue medications for asthma in individuals with no clear clinical benefit or without confirmation of reversible airflow limitation or airway hyperresponsiveness with objective testing [23]. Following these recommendations, there was insufficient documentation of subjective or objective response to empiric controller therapy within the primary care EMR to support response to empiric therapy as objective proof of confirmed asthma in our study.

#### Use of PFTs for Diagnosis and Monitoring

To determine the appropriate use of objective lung function measures for diagnosis and monitoring, key recommendations were used from CTS guidelines. Within the current guidelines, objective confirmation of diagnosis status is recommended for all new asthma diagnoses in patients aged 6 years and older [3]. Specific criteria are reversible airflow obstruction on spirometry, variable airflow obstruction over time, or evidence of airway hyperresponsiveness on a challenge test such as methacholine or exercise-induced asthma challenge [3]. Additionally, a key parameter for achieving CTS guideline adherence includes measuring lung function (spirometry or peak expiratory flow) at follow-up visits, as part of ongoing assessment of asthma control [3,24].

### Statistical Analysis

#### Overview

Descriptive statistics, including frequency and percentages, were performed on data elements extracted from manual chart abstraction and PAAF extraction. Univariate inferential statistics were performed on validated primary care performance indicators and data elements pertaining to asthma diagnosis and management [18]. Chi-square tests were performed on categorical variables, and unpaired 2-tailed *t* tests were performed on continuous numerical variables. Statistical significance was set as *P*<.05 for all analyses. All statistical analysis was performed on Microsoft Excel and GraphPad PRISM software.

#### Sample Size Calculation

To ensure the statistical power of primary outcomes, 2 sample size calculations were performed. For the use of objective lung function measures to confirm an asthma diagnosis, to achieve a power of 80%, at a significance of .05, from a baseline 25% of patients with objectively confirmed asthma [22], and a clinically meaningful effect of 20%, we needed 67 patients in the baseline cohort and 134 in the postimplementation cohort. For the documentation of diagnosis status in EMRs, to achieve a power of 80%, at a significance of .05, from a baseline 10% of patients with documentation diagnosis status [22], and a clinically meaningful effect of 20%, we needed 48 patients in the baseline cohort and 96 in the postimplementation cohort. Therefore, we aimed for over 67 patients in the baseline cohort and 134 in the postimplementation to ensure adequate statistical power.

## Results

### Sample Derivation and Characteristics

**Figure 2.**
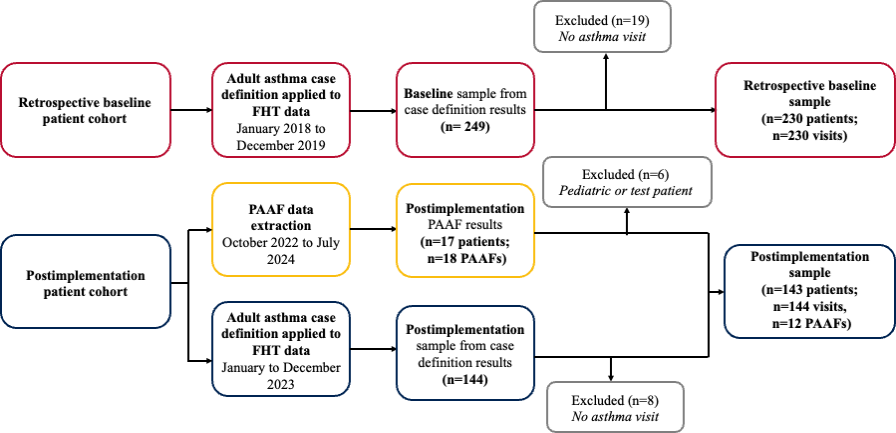
Sample derivation. FHT: Family Health Team; PAAF: Provider Asthma Assessment Form.

A total of 373 patients were deemed eligible in the final analysis of this study. The sample derivation is displayed in Figure 2. In the retrospective baseline patient cohort, 230 patients were included. In the postimplementation patient cohort, a total of 143 patients were included, of whom the PAAF was used in 12. A total of 27 patients were excluded from manual chart abstraction (retrospective baseline: n=19 and postimplementation: n=8), as they did not meet eligibility for having an asthma visit within the study period. A total of 6 patients (6 PAAFs) were excluded, as they were identified as either a pediatric patient or a test patient chart (ie, a chart generated for training and programming purposes). There were 7 new patients from the PAAF data extraction, and 5 patients overlapped with the postimplementation chart abstraction.

Table 1 displays the patient characteristics of included patients from the retrospective baseline and postimplementation. No significant differences were found between the 2 groups, except for a significantly higher percentage of patients diagnosed with diabetes in the postimplementation cohort (retrospective baseline: n=14, 6.1% vs postimplementation: n=18, 12.6%; *P*=.04). Both the mean age (retrospective baseline: mean 46.9, SD 17.4; postimplementation: mean 49.7, SD 17.5; *P*=.14) and BMI (kg/m^2^) were similar between groups (retrospective baseline: mean 30.0, SD 8.4; postimplementation: mean 31.3, SD 7.7; *P*=.14). There was a high percentage of female patients compared to male patients (sex assigned at birth) in both cohorts (retrospective baseline: n=135, 58.7% female vs postimplementation: n=90, 62.9% female; *P*=.45). There was an observed high percentage of comorbidities with both groups having over 45% of patients with ≥4 comorbidities (retrospective baseline: n=107, 46.5% vs postimplementation: n=65, 45.5%; *P*=.60). The most common comorbidity among both groups was allergies (retrospective baseline: n=187, 81.3% vs postimplementation: n=113, 79%; *P*=.59).

**Table 1.** Patient demographics and comorbidities.

Characteristic	Retrospective baseline (n=230)	Postimplementation (n=143)	*P* value
Age (years), mean (SD)	46.9 (17.4)	49.7 (17.5)	.14
Sex assigned at birth, n (%)			.45
Male	95 (41.3)	53 (37.1)	
Female	135 (58.7)	90 (62.9)	
BMI (kg/m^2^), mean (SD)	30 (8.4)	31.3 (7.7)	.13
Number of comorbidities, n (%)			.60
0	9 (3.9)	5 (3.5)	
1	29 (12.6)	12 (8.4)	
2	29 (12.6)	23 (16.1)	
3	56 (24.3)	38 (26.6)	
≥4	107 (46.5)	65 (45.5)	
Comorbidities, n (%)			
Allergic rhinoconjunctivitis	118 (51.3)	87 (60.8)	.09
Allergies	187 (81.3)	113 (79)	.59
Anaphylaxis	30 (13)	14 (9.8)	.41
Atopic dermatitis	13 (2.2)	7 (4.9)	.82
Asthma-COPD^a^ overlap	5 (2.2)	7 (4.9)	.23
COPD	6 (2.6)	3 (2.1)	.99
Diabetes	14 (6.1)	18 (12.6)	.04^b^
Eczema or hives or uticaria	33 (14.3)	12 (8.4)	.10
Obesity	95 (41.3)	58 (40.6)	.91
GERD^c^	68 (29.6)	46 (32.2)	.64
Psychopathologies	103 (44.6)	69 (48.2)	.67
OSA^d^	52 (22.6)	31 (21.7)	.90
Rhinitis or nasal polyposis or sinusitis	26 (11.3)	18 (12.6)	.74

a COPD: chronic obstructive pulmonary disease.

b *P*<.05.

c GERD: gastroesophageal reflux disease.

d OSA: obstructive sleep apnea.

### Diagnosis Status and Monitoring With PFTs

In total, only 31.3% (n=72) of patients in the retrospective baseline and 23.8% (n=34) in the postimplementation cohorts were classified as having confirmed asthma (Table 2). The most common method of diagnosis in both groups was evidence of reversible airflow obstruction on spirometry (retrospective baseline: n=36, 50% vs postimplementation: n=21, 61.8%). The second most common method was a positive or borderline methacholine challenge test (retrospective baseline: n=29, 40.3% vs postimplementation: n=6, 17.6%). A higher proportion of confirmed patients with asthma in the postimplementation cohort had their asthma diagnosed by a specialist (respirologist, allergist, or internal medicine) without documented evidence of objective measures (postimplementation: n=4, 11.8% vs retrospective baseline: n=7, 9.7%). A visual representation of suspected versus confirmed asthma can be appreciated in Multimedia Appendix 1.

Table 3 displays results pertaining to monitoring with PFTs. The most noteworthy finding was that PFTs were requested by physicians in a significantly higher proportion of patients in the postimplementation period (postimplementation: n=70, 49% vs retrospective baseline: n=71, 30.9%; *P*<.001). A nonsignificant higher proportion of patients in the retrospective baseline cohort had been monitored with PFTs within 12 months before the documented encounter (retrospective baseline: n=67, 29.1% vs postimplementation: n=28, 19.6%; *P*=.05). However, a nonsignificantly higher proportion of patients had been monitored with PFTs either 12 months before or after the documented encounter (postimplementation: n=50, 35% vs retrospective baseline: n=71, 30.9%; *P*=.43). A visual representation of PFTs completed versus PFTs requested by providers can be appreciated in Multimedia Appendix 2.

**Table 2.** Diagnosis status.

Diagnosis status	Retrospective baseline (n=230), n (%)	Postimplementation (n=143), n (%)	*P* value
Suspected	158 (68.7)	109 (76.2)	.17
Confirmed	72 (31.3)	34 (23.8)	.17
Reversible airflow obstruction (spirometry)^a^	36 (50)	21 (61.8)	
Positive or borderline methacholine challenge^a^	29 (40.3)	6 (17.6)	
Positive exercise challenge^a^	0 (0)	2 (5.9)	
Peak expiratory flow variability^a^	0 (0)	1 (2.9)	
Specialist diagnosis alone^a^	7 (9.7)	4 (11.8)	
Excluded	1 (0.4)	0 (0)	.17

a Percentages based on number of confirmed patients.

**Table 3.** Pulmonary function testing.

Pulmonary function testing	Retrospective baseline (n=230), n (%)	Postimplementation (n=143), n (%)	*P* value
PFTs^a^ requested in the last 12 months	71 (30.9)	70 (49)	<.001^b^
Monitored with PFTs in the last 12 months	67 (29.1)	28 (19.6)	.05
Monitored with PFTs within 12 months before or after documented visit	71 (30.9)	50 (35)	.43

a PFT: pulmonary function test.

b *P* value with significance <.05.

### Medications

Table 4 summarizes the asthma medications documented in the charts of patients at the time of the asthma visit included in the study. Two primary significant differences were found. In the postimplementation group, 21.7% (n=31) of patients were on single inhaler reliever and controller therapy (single inhaler therapy [SIT]) compared to 0.9% (n=2) on SIT in the retrospective baseline cohort (*P*<.001). Similarly, a significantly greater percentage of patients (n=36, 25.2%) in the postimplementation groups were on ICS/long-acting β-2 agonist (LABA) controller therapy compared to the retrospective baseline (n=34, 14.8%; *P*=.01). No other significant differences were found between groups. Nonsignificant trends include a 5.6% decrease in systemic steroid use and a 5% decrease in adherence issues after implementation. A summary of reliever, ICS, second controller, systemic steroid use, and SIT can be found in Multimedia Appendix 3.

**Table 4.** Medications.

Medication	Retrospective baseline (n=230), n (%)	Postimplementation (n=143), n (%)	*P* value
Reliever	186 (80.9)	125 (87.4)	.12
SABA^a^	182 (79.1)	116 (81.1)	.69
SAMA^b^	12 (5.2)	4 (2.8)	.31
Controller			
ICS^c^	128 (55.7)	92 (64.4)	.11
Single inhaler therapy	2 (0.9)	31 (21.7)	<.001^d^
Second controller	81 (35.2)	63 (44.1)	.10
ICS/LABA^e^	34 (14.8)	36 (25.2)	.01^d^
ICS/LAMA^f^/LABA	0 (0)	2 (1.4)	.15
LAMA	12 (5.2)	5 (3.5)	.61
LABA	3 (1.3)	2 (1.4)	>.99
LTRA^g^	23 (10)	9 (6.3)	.26
ICS dosage^h^			.49
Low	57 (44.5)	50 (54.3)	
Medium	49 (38.3)	32 (34.8)	
High	26 (20.3)	16 (17.4)	
Biologic	4 (1.7)	2 (1.4)	>.99
Omalizumab^i^	4 (100)	1 (50)	
Tezepelumab^i^	0 (0)	1 (50)	
Systemic corticosteroids used within the last year	58 (25.2)	28 (19.6)	.26
Adherence issues known or suspected	47 (20.4)	22 (15.4)	.27
Asthma medication prescribed or changed at visit	130 (56.5)	78 (54.5)	.75

a SABA: short-acting β-2 agonist.

b SAMA: short-acting muscarinic antagonist.

c ICS: inhaled corticosteroid.

d *P* values with significance <.05.

e LABA: long-acting β-2 agonist.

f LAMA: long-acting muscarinic antagonist.

g LTRA: leukotriene receptor antagonist.

h ICS percentages based on a number of patients on an ICS.

i Biologic percentages based on a number of patients on a biologic.

### Asthma Control

At least 1 CTS control parameter was documented in a high percentage of patients in both groups (retrospective baseline: n=226, 98.3% vs postimplementation: n=138, 96.5%; *P=*.31; Table 5). A significantly higher percentage of postimplementation patients were on an ICS if their asthma was uncontrolled (postimplementation: n=69, 62.2% vs retrospective baseline: n=100, 43.5%; *P=*.002). Furthermore, a significantly higher number of CTS asthma control parameters were documented after implementation (postimplementation: mean 2.5, SD 1.3 vs retrospective baseline: mean 2.2, SD 1.2; *P*=.02). A summary of asthma control documentation is represented in Multimedia Appendix 4.

**Table 5.** Asthma control: baseline versus postimplementation.

Asthma control	Retrospective baseline (n=230)	Postimplementation (n=143)	*P* value
≥1 Asthma control parameter documented, n (%)	226 (98.3)	138 (96.5)	.31
Asthma controlled at visit, n (%)	30 (13)	28 (19.6)	.11
Asthma uncontrolled at visit, n (%)	195 (84.8)	111 (77.6)	.10
Uncontrolled asthma on an ICS^a^^,^^b^	100 (43.5)	69 (62.2)	.002^c^
Number of asthma control parameters documented, mean (SD)	2.2 (1.2)	2.5 (1.3)	*.*02^c^

a Uncontrolled asthma on ICS percentages and *P* value based on a total number of patients with uncontrolled asthma.

b ICS: inhaled corticosteroid.

c *P* values with significance <.05.

### Health Services Use, Asthma Management, and PC-APIs

Table 6 summarizes the health services use and management between cohorts. A significantly higher proportion of barriers were addressed during the documented asthma visit in the postimplementation group compared to the baseline (postimplementation: n=24, 16.8% vs retrospective baseline: n=11, 4.8%; *P*<.001). No other significant differences were found. Although not statistically significant, the proportion of recent emergency department visits in <1 year decreased by 4% in the postimplementation group (postimplementation: n=16, 11.2% vs retrospective baseline: n=35, 15.2%; *P=*.21). An Asthma Action Plan (AAP) was provided or revised or reviewed more often (4.1% more) in the postimplementation period (*P*=.15). Several PC-APIs from both cohorts are represented in Table 7.

**Table 6.** Health services use and asthma management.

Health services use and asthma management	Retrospective baseline (n=230), n (%)	Postimplementation (n=143), n (%)	*P* value
Health services use			
ED^a^ ever for asthma	75 (32.6)	50 (35)	.65
Recent ED for asthma (<1 year)	35 (15.2)	16 (11.2)	.21
Hospitalized ever for asthma	33 (14.3)	21 (14.7)	>.99
Recent hospitalization for asthma (<1 year)	6 (2.6)	0 (0)	.09
Specialist involved in care (respirologist, internal medicine, or allergist)	64 (27.8)	36 (25.2)	.63
Exacerbation<1 year	109 (47.4)	60 (42)	.34
Smoking status			
Current	34 (14.8)	21 (14.7)	.98
Smoking cessation addressed^b^	19 (55.9)	14 (66.7)	.57
Past	63 (27.4)	41 (28.7)	.98
Care, management, and education			
Device technique assessed	11 (4.8)	8 (5.6)	.81
Triggers addressed	176 (76.5)	109 (76.2)	>.99
Environmental controls discussed	29 (12.6)	12 (8.4)	.24
Barriers addressed	11 (4.8)	24 (16.8)	<.001
Referred to an asthma education program or CRE^c^	8 (3.5)	8 (5.6)	.43
Primary care physician suspected severe asthma	6 (2.6)	7 (4.9)	.26
Referral to a specialist for suspected severe asthma	4 (1.4)	5 (3.5)	.31
Abstractor suspected severe asthma^d^	10 (4.3)	10 (7)	.34
Asthma Action Plan provided or revised or reviewed	13 (5.7)	14 (9.8)	.15

a ED: emergency department.

b Smoking cessation addressed percentages based on a number of current smokers.

c CRE: Certified Respiratory Educator.

d Abstractor suspected severe asthma based on Canadian Thoracic Society criteria and the severe asthma algorithm.

**Table 7.** Primary Care—Asthma Performance Indicators.

Primary Care—Asthma Performance Indicators	Retrospective baseline (n=230), n (%)	Postimplementation (n=143 patients), n (%)	*P* value
Referrals to an asthma education program or CRE^a^	8 (3.5)	8 (5.6)	.43
Pulmonary function monitoring in the last 12 months	71 (30.9)	50 (35)	.43
Patients with uncontrolled asthma on an ICS^b^	100 (43.5)	69 (62.2)	.002
Well-controlled asthma	30 (13)	28 (19.6)	.11
ED^c^ visits for asthma <1 year	35 (15.2)	16(11.2)	.21
Objectively confirmed asthma	72 (31.3)	34 (23.8)	.12
Smoking cessation addressed	19 (8.3)	14 (9.8)	.57
Asthma exacerbations	109 (47.4)	60 (42)	.34
Inhaler technique monitoring	11 (4.8)	8 (5.6)	.81
Asthma Action Plan	3 (15.7)	14 (9.8)	.15

a CRE: Certified Respiratory Educator.

b ICS: inhaled corticosteroid.

c ED: emergency department.

### Use of the PAAF Versus Manual Chart Abstraction

Overall, 12 PAAFs were used during asthma encounters with 11 patients total in the postimplementation period. When the PAAF was used, diagnosis status was documented 100% (n=12) of the time. When the PAAF was not used, a detailed manual chart review was needed to determine the diagnosis status.

Asthma control documentation was compared between PAAFs used and manual chart abstraction from EMR encounter notes (Table 8). In terms of asthma control, the average number of CTS asthma control parameters documented was significantly higher when the PAAF was used (PAAF: mean 5.4, SD 1.9 vs manual chart abstraction: mean 2.3, SD 1.2; *P*<.001). Asthma control was documented significantly more often in manual chart abstractions (PAAF: n=7, 58.3% vs manual chart abstraction: n=360, 98.4%; *P*<.001). Finally, there was a significantly higher proportion of patients whose asthma was uncontrolled at the time of the visit in manual chart abstractions (manual chart abstraction: n=302, 82.5% vs PAAF: n=6, 50%; *P*=.01).

**Table 8.** Asthma control: manual chart abstractions versus Provider Asthma Assessment Form (PAAF).

Asthma control	Manual chart abstraction (n=366)	PAAF (n=12)	*P* value^a^
≥1 Asthma control parameter documented, n (%)	360 (98.4)	7 (58.3)	<.001
Asthma uncontrolled at visit, n (%)	302 (82.5)	6 (50)	.01
Number of asthma control parameters documented, mean (SD)^b^	2.3 (1.2)	5.4 (1.9)	<.001

a All values were significant (*P*<.05).

b Average number of asthma control parameters was based on asthma visits where asthma control was documented.

## Discussion

### Summary of Main Findings

Following a multifaceted intervention of implementing the PAAF in this primary care practice, there were substantial improvements in asthma-specific documentation and adherence to key evidence-based recommendations for care. There was an 18.1% increase in the number of PFTs requested after the implementation of the PAAF. This did not translate to the number of confirmed asthma cases, presumably due to the impacts of the COVID-19 pandemic on PFT wait times. Furthermore, a highly significant and clinically relevant increase was noted in the proportion of patients prescribed SIT after implementation of the PAAF (postimplementation: n=31, 21.7% vs baseline: n=2, 0.9%), and a significantly greater proportion of patients were on ICS/LABA controller therapy after implementation. In general, a higher percentage of patients were on reliever, ICS, and second controller therapy after implementation, and a smaller proportion had received systemic corticosteroids in the last year. Asthma control was highly documented in both cohorts, but significantly more CTS asthma control parameters were documented after implementation, and a higher proportion of patients with uncontrolled asthma were prescribed ICS therapy. A greater percentage of patients had barriers addressed at the asthma encounter after implementation compared to baseline (postimplementation: n=24, 16.8% vs baseline: n=11, 4.8%). There were no meaningful changes in specialist referrals due to suspected SA. Although it is not possible to discern causality for observed changes based on this study, overall, care as assessed by key PC-APIs showed improvement in the postimplementation cohort.

### Interpretation of Main Findings

#### Diagnosis Status and Monitoring With PFTs

Overall, the proportion of patients in this family practice with confirmed asthma in both cohorts (~25%‐30% of patients) is consistent with previous literature [22]. The finding of PFTs completed or requested within 12 months of the encounter date is consistent with similar literature in Ontario [21]. A study by Gershon et al [21] found that <50% of Ontarian people underwent spirometry within 1 year before and 2.5 years after a new diagnosis of asthma. Our study found that 49% (n=70) of postimplementation patients and 30.9% (n=71) of baseline patients had a PFT requested. Thus, less than 50% of patients in this study had been monitored with a PFT within 12 months of an asthma encounter with their primary care provider. However, it is important to note that our study did not differentiate new diagnoses from previous diagnoses for the purposes of analysis. Additionally, our study found an 18.1% significant increase in PFT requests in the postimplementation cohort versus the retrospective baseline. However, this did not yet translate to PFTs completed in the postimplementation group. The reduced number of patients in postimplementation with confirmed asthma (baseline: n=72, 31.3% vs postimplementation: n=34, 23.8%) may in part be attributed to the backlog of requests and limited access to PFTs due to the COVID-19 pandemic. As previously stated, a greater proportion of PFTs were requested after implementation but not completed 12 months prior to the asthma encounter date. It is evident that a key asthma care gap persists at this family practice, as there is limited confirmation of an asthma diagnosis and monitoring asthma with PFTs. It would be prudent in the future to determine the barriers to confirmation of diagnosis status and potentially incorporate diagnosis status as a required data element to be documented within primary care EMRs, such as including validated PRESTINE data elements for asthma diagnosis [12]. In addition, it would be beneficial to assess and address the current availability, associated costs, and accessibility of PFTs in Canadian primary care settings.

#### Medications

It was interesting to see the significant increase in SIT in the postimplementation cohort. In recent CTS guidelines, SIT consists of pharmacologic management with budesonide-formoterol inhaler both as a PRN (as needed) reliever and controller therapy [3]. In the Global Initiative for Asthma (GINA) report 2024, SIT (known in the GINA report as maintenance-and-reliever therapy) is recommended treatment for GINA steps 3‐4 [7]. Hence, there was increased adherence with this guideline recommendation in the postimplementation period. There were also significantly more patients on an ICS/LABA inhaler after implementation. It is a CTS guidelines recommendation that adults with asthma not achieving adequate control on a low-dose ICS be escalated to ICS/LABA therapy [3]. Hence, this can be seen as greater adherence to CTS guidelines as well. Overall, there was increased use of reliever, ICS, and second controller therapy after implementation. There was also a reduction in systemic corticosteroid use in the postimplementation period (5.6% decrease). The reduction in systemic corticosteroid use may be secondary to appropriate increased use of ICS therapies (ICS and ICS/LABA) [3].

#### Asthma Control

CTS guidelines recommend that asthma control be assessed at each clinical encounter [3]. There was a high degree of compliance with this recommendation in both cohorts, as asthma control was documented in over 96% of asthma encounters. Guidelines also outline 9 asthma control parameters that aid in determining whether asthma is well-controlled at the encounter [3]. In this study, control was documented in a higher average number of encounters in the postimplementation period. However, less than 3 asthma control parameters were documented in both groups. Asthma control would be more accurately assessed during asthma encounters if a greater number of control parameters were assessed [3]. When all control parameters are not documented or assessed, the classification of asthma control could be over- or underestimated.

#### Severe Asthma

The clinical decision support component of the PAAF includes an embedded SA algorithm, which aids in the recognition and referral of uncontrolled asthma and/or SA. This functionality of the PAAF directly integrates key messaging from a recent CTS position statement on SA and the Pan-Canadian standards for SA in EMRs [6,17]. In primary care, key messaging focuses on improved recognition of uncontrolled asthma and/or SA and increasing referrals for specialized evaluation and asthma education. Thus, the SA outcomes for this study pertained to the recognition of SA by providers and referrals to specialist care.

In terms of identification of SA, primary care physicians suspected SA in 2.6% (n=6) of baseline patients and 4.9% (n=7) of postimplementation patients. In contrast, the abstractor suspected SA based on the SA algorithm [17] in 4.3% (n=10) of baseline and 7% (n=10) of postimplementation. SA was suspected more often in both cohorts based on the SA algorithm compared to primary care providers without the use of the algorithm [17]. This represents a persistent care gap for SA, which could be improved by broader adoption of the PAAF.

#### Health Services Use, Asthma Education, and PC-APIs

Overall, there was limited adherence to CTS guidelines for best-practice asthma education in both cohorts. The most common aspect of asthma education addressed at each primary care encounter was asthma triggers, as triggers were addressed in over 76% of asthma encounters. However, various core components of effective asthma education were not addressed in this patient population.

CTS guidelines highlight the importance of providing self-management education including a written AAP, discussing environmental controls, and referring patients to asthma education programs when appropriate [3]. AAPs were provided, revised, or reviewed in less than 10% of encounters, and environmental control was discussed in less than 13%. Notably, less than 6% of patients in both cohorts were referred to an asthma education program or Certified Respiratory Educator (CRE), despite having access to an on-site CRE each month as part of the Primary Care Asthma Program [25]. There is strong evidence that community evidence–based asthma care programs improve clinical outcomes, including a decreased risk of asthma exacerbations and urgent health services use [25]. However, this resource was not effectively used in baseline or postimplementation. It would be warranted to further investigate in the future the reasoning behind the lack of use of this resource.

Overall, care as assessed by key PC-APIs showed tendencies toward improvement in the postimplementation cohort, although most did not achieve statistical significance. A significant finding was that an increased proportion of patients with uncontrolled asthma were on an ICS in the postimplementation. Additionally, a positive trend included a 5% reduction in known or suspected adherence issues with pharmacologic therapy after implementation. CTS guidelines [3] and the GINA report [7] emphasize the fundamental importance of appropriate ICS therapy in achieving asthma control for individuals with uncontrolled asthma and preventing future risk of exacerbations. Thus, these findings demonstrated increased adherence with evidence-based guidelines.

Furthermore, in the postimplementation period, a greater proportion of patients received AAPs, had inhaler technique monitoring, smoking cessation addressed, well-controlled asthma, PFT monitoring in the last 12 months, and referrals to an asthma education program or CRE. There were also decreased emergency department visits in the previous year and recent asthma exacerbations after implementation. Overall, the positive trends suggest that implementation of the PAAF, either the tool itself or the implementation strategies used in the study, may be associated with improvements in asthma management and better adherence with best-practice guidelines. However, we did not see a noteworthy change in one of the primary outcomes, specialist referral of patients with suspected SA. Low uptake of the PAAF meant that the study was underpowered to detect significance in specialist referral for suspected SA.

#### Use of the PAAF

A comparison between the use of the PAAF to document an asthma encounter compared to manual chart abstraction was conducted. When the PAAF was used, asthma diagnosis status (either suspected or confirmed) was documented 100% of the time. Hence, the use of the PAAF would aid in addressing one of the key asthma care gaps in primary care, confirming asthma through objective measures [3,19,26].

Additionally, a significantly higher proportion of asthma control parameters were documented when the PAAF was used compared to encounter notes used for manual chart abstraction. Compared to current EMR primary documentation of asthma management in encounter notes, the use of the PAAF eases access to pertinent medical history as well as documentation of diagnosis status and asthma control.

#### Provider Uptake

One of the main challenges of this study was the uptake of the PAAF in the clinic and the participation of providers throughout the intervention. This could be due to many factors, such as change management issues (diffusion of innovations theory) [27], the implementation model did not address sufficient determinants of behavior to effect change (Theoretical Domains Framework) [28,29], or insufficient time in the clinic for providers to adopt a new tool. Thus, the perceived utility, provider satisfaction, and barriers and enablers for the use of the form are investigated in another study. However, future studies involving the PAAF could use various other methods to increase uptake in the clinic.

Globally, there is evidence supporting the various benefits of multidisciplinary primary health care teams to meet the changing demands of the health care system [30-32]. Hence, there is an increased occurrence of and need for task shifting from physicians to allied health care professionals [30-32]. Particularly in Canada, there are a growing number of unattached patients with a survey in 2022 finding that ~22% of patients across Canada reported not having a family physician or nurse practitioner [33]. The shift toward multidisciplinary teams would aid in alleviating administrative burden [32], aid in chronic disease management (ie, asthma) [34], and allow for an increased number of patients to be connected to comprehensive primary health care [35]. Therefore, it would be reasonable to design a multifaceted, multidisciplinary intervention to implement the PAAF in primary care practice to increase user uptake. This would be particularly relevant if the PAAF was used as part of a broader asthma intervention involving spirometry testing and asthma education in primary care [34].

#### Comparison to the Literature

In comparison to other EMR-integrated asthma KT tools, our results were similar. Our findings can be compared to 2 primary care EMR KT tools. The electronic asthma management system was found to be an efficacious tool, particularly increasing AAP delivery, assessment of control, and escalation of pharmacologic therapy after the intervention [15,36]. However, similar to our study, there was limited user uptake beyond the scope of the initial study period [15]. A similar result was found during the evaluation of the breathe (University Health Network) mobile app, with limited user uptake [14]. It is important to distinguish that these e-Tools were patient-facing tools with EMR integration for providers to view results entered by patients. The PAAF, breathe app, and electronic asthma management system were all found to be efficacious tools for asthma management, but long-term engagement with the tools by primary care providers remains low.

### Limitations

The main limitation of this study was the limited uptake of the PAAF in the primary care practice. Therefore, the sample size was limited, potentially impacting the power of our study. As can be seen from the CPCSSN case definition search, there were 136 visits that would have been eligible for the PAAF to be used in the postimplementation time frame. However, it was only used 12 times throughout the study duration. The perceived utility, provider satisfaction, and barriers and enablers to PAAF use are the focus of another study.

Furthermore, it is important to note that the results of this study can only be interpreted in the context of the broader multifaceted intervention encompassing not only the PAAF tool but also the various implementation strategies used as part of the training and orientation to the tool. Further studies are needed to differentiate the specific impact of the tool itself, rather than the global strategies used as part of the implementation of the PAAF in a primary care setting.

It is important to note that it is difficult to discern causality for the changes seen in asthma management practice in this study. As this study was conducted at an academic primary care practice, the resident physicians involved with patient care receive additional instruction and education on effective asthma management during their academic instructional time. As well, there was a concurrent quality improvement project focusing on the Choosing Wisely Canada climate change and inhaler recommendations for patients with asthma [37]. This quality improvement initiative aimed to decrease the number of patients on pressurized metered dose inhalers and increase the number of patients on dry-powder inhalers, which may have influenced the proportion of patients on SIT (which is commonly a dry-powder inhaler). Therefore, it is important to recognize that this initiative may have increased the focus on asthma management in the primary care practice, beyond the implementation efforts for the PAAF.

Additionally, the percentage of postimplementation patients with a PFT in the last 12 months was likely impacted due to a persistent backlog in the Pulmonary Function Lab at Kingston Health Sciences Centre (1 of 2 centers for PFT testing in Kingston) due to the COVID-19 pandemic. Hence, this study may not reflect a complete picture of PFT monitoring in baseline compared to postimplementation. In an effort to address this limitation, the number of requested PFTs within 12 months of the documented encounter was included in the analysis.

It is pertinent to recognize the inherent limitations of manual chart abstraction as well. Data elements collected are limited by the information providers document within the chart and encounter notes; thus, the documentation may not fully capture all aspects of care discussed during an asthma visit.

### Conclusions

Following the multifaceted intervention of implementing the PAAF in this primary care practice, there were significant improvements in asthma-specific documentation and adherence to key evidence-based recommendations for care. The use of the PAAF increased asthma visit–specific documentation, such as clearly documenting diagnosis status and asthma control parameters. However, asthma care gaps such as the underuse of PFTs for diagnosis and monitoring, asthma education, and addressing reasons for poor asthma control were still common in this study. Future directions should involve evaluating the impact of the PAAF after widespread implementation in primary care settings and investigating methods to increase user uptake of the PAAF.

## Supplementary material

10.2196/74043 Multimedia Appendix 1 Confirmed versus suspected asthma.

10.2196/74043 Multimedia Appendix 2 Pulmonary function tests completed versus requested. *P<.001.

10.2196/74043 Multimedia Appendix 3 Summary of prescribed asthma medications. **P<.001.

10.2196/74043 Multimedia Appendix 4 Asthma control documentation.

## Abbreviations

AAP: Asthma Action Plan
CPCSSN: Canadian Primary Care Sentinel Surveillance Network
CRE: Certified Respiratory Educator
CTS: Canadian Thoracic Society
e-Tool: electronic tool
EMR: electronic medical record
FHT: Family Health Team
GINA: Global Initiative for Asthma
*ICD-9*: *International Classification of Diseases, Ninth Revision*
ICS: inhaled corticosteroid
KT: Knowledge Translation
LABA: long-acting β-2 agonist
PAAF: Provider Asthma Assessment Form
PC-API: Primary Care—Asthma Performance Indicator
PFT: pulmonary function test
PRESTINE: Pan-Canadian Respiratory Standards Initiative for Electronic Health Records
SA: severe asthma
SIT: single inhaler therapy
