# Supplementary figures and images for: Impact of a Novel Electronic Medical Record–Integrated Electronic Form (Provider Asthma Assessment Form) and Severe Asthma Algorithm in Primary Care: Single-Center, Pre- and Postobservational Study
Source: JMIR Form Res. 2025 Jun 25;9:e74043. doi: 10.2196/74043 (PMC12242708; doi:10.2196/74043)

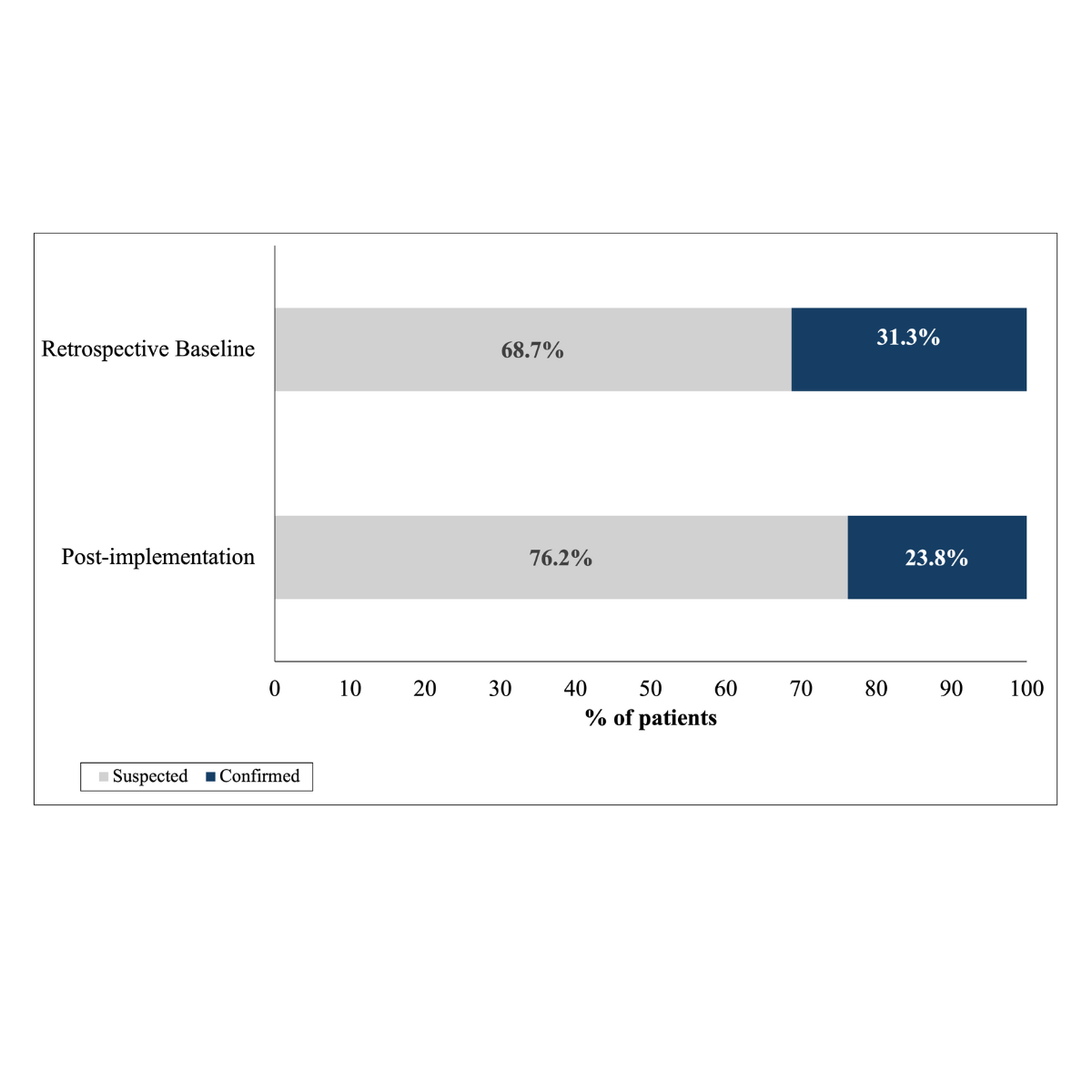

Supplement: Multimedia Appendix 1 [file formative-v9-e74043-s001.png]

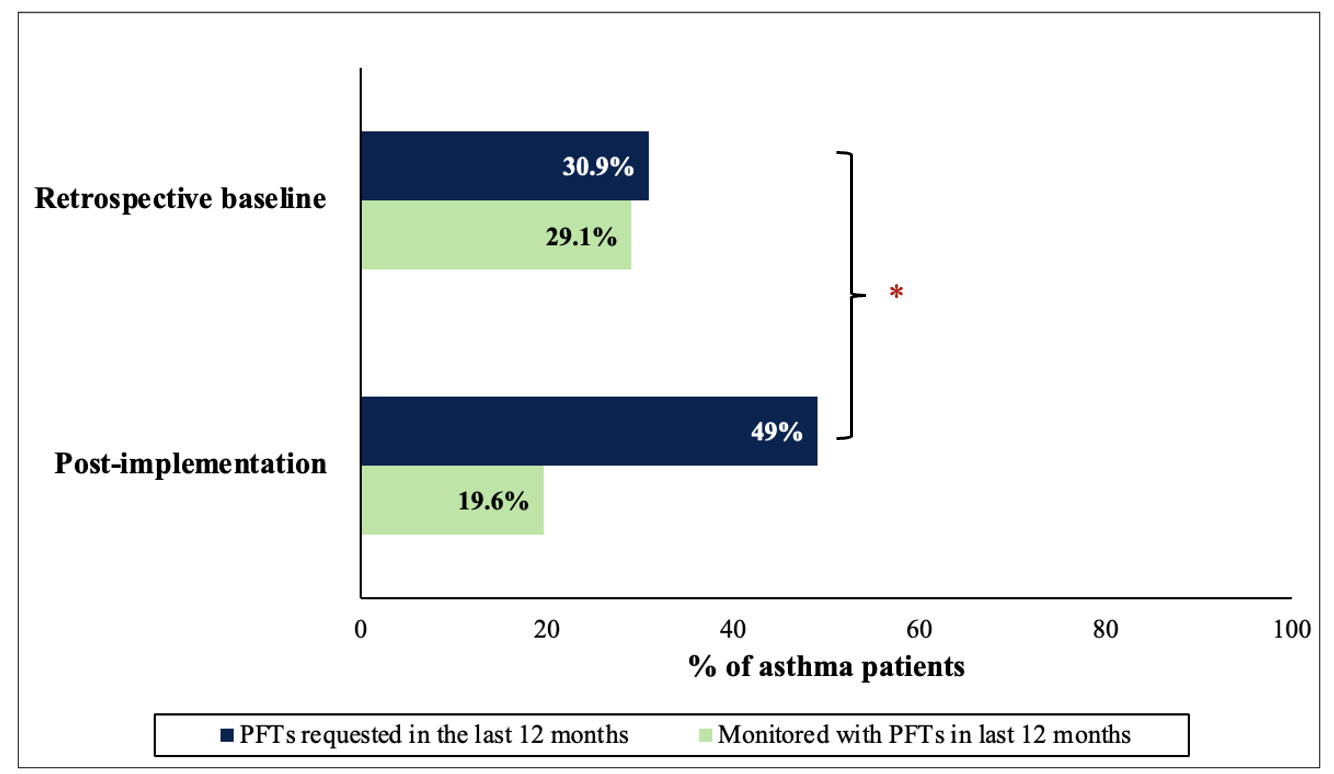

Supplement: Multimedia Appendix 2 [file formative-v9-e74043-s002.png]

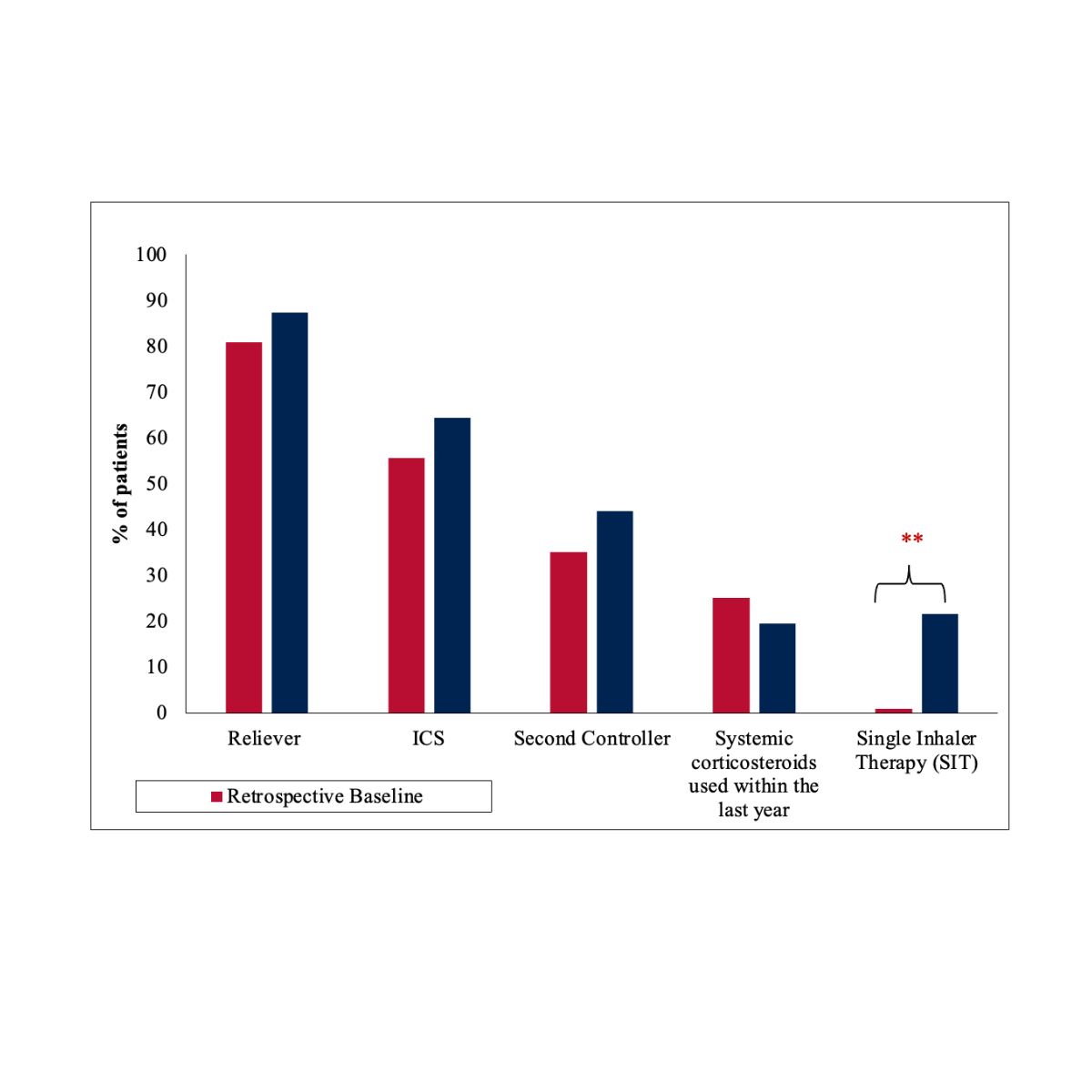

Supplement: Multimedia Appendix 3 [file formative-v9-e74043-s003.png]

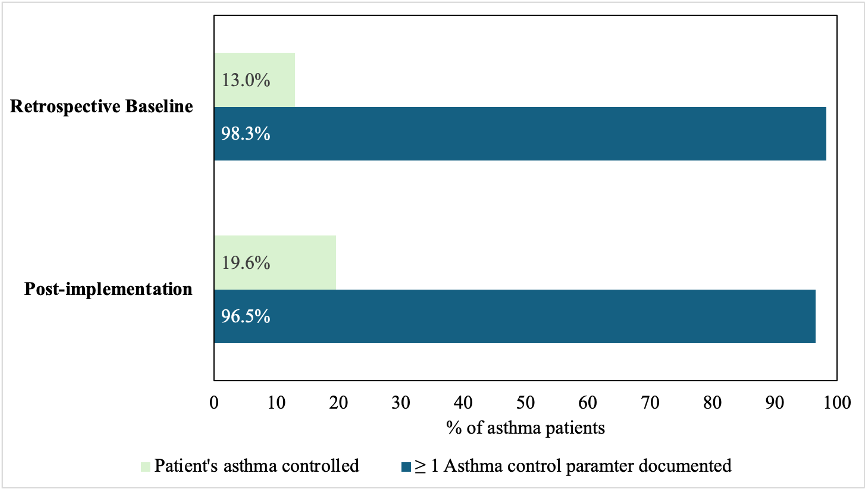

Supplement: Multimedia Appendix 4 [file formative-v9-e74043-s004.png]
